# A multispeaker dataset of raw and reconstructed speech production real-time MRI video and 3D volumetric images

**DOI:** 10.1038/s41597-021-00976-x

**Published:** 2021-07-20

**Authors:** Yongwan Lim, Asterios Toutios, Yannick Bliesener, Ye Tian, Sajan Goud Lingala, Colin Vaz, Tanner Sorensen, Miran Oh, Sarah Harper, Weiyi Chen, Yoonjeong Lee, Johannes Töger, Mairym Lloréns Monteserin, Caitlin Smith, Bianca Godinez, Louis Goldstein, Dani Byrd, Krishna S. Nayak, Shrikanth S. Narayanan

**Affiliations:** 1 Ming Hsieh Department of Electrical and Computer Engineering, Viterbi School of Engineering, University of Southern California, Los Angeles, California USA; 2 Department of Linguistics, Dornsife College of Letters, Arts and Sciences, University of Southern California, Los Angeles, California USA; 3 Department of Linguistics, California State University Long Beach, Long Beach, California USA

**Keywords:** Translational research, Biomedical engineering, Electrical and electronic engineering

## Abstract

Real-time magnetic resonance imaging (RT-MRI) of human speech production is enabling significant advances in speech science, linguistics, bio-inspired speech technology development, and clinical applications. Easy access to RT-MRI is however limited, and comprehensive datasets with broad access are needed to catalyze research across numerous domains. The imaging of the rapidly moving articulators and dynamic airway shaping during speech demands high spatio-temporal resolution and robust reconstruction methods. Further, while reconstructed images have been published, to-date there is no open dataset providing raw multi-coil RT-MRI data from an optimized speech production experimental setup. Such datasets could enable new and improved methods for dynamic image reconstruction, artifact correction, feature extraction, and direct extraction of linguistically-relevant biomarkers. The present dataset offers a unique corpus of 2D sagittal-view RT-MRI videos along with synchronized audio for 75 participants performing linguistically motivated speech tasks, alongside the corresponding public domain raw RT-MRI data. The dataset also includes 3D volumetric vocal tract MRI during sustained speech sounds and high-resolution static anatomical T2-weighted upper airway MRI for each participant.

## Abstract (summary)

**Table:** 

Measurement(s)	Speech • vocal tract
Technology Type(s)	magnetic resonance imaging
Factor Type(s)	age • sex • native/non-native speaker
Sample Characteristic - Organism	Homo sapiens

Machine-accessible metadata file describing the reported data: 10.6084/m9.figshare.14892921

## Background & Summary

Human upper airway functions such as swallowing, breathing, and speech production are the result of a well-coordinated choreography of various mobile soft tissue and muscular structures such as the tongue, lips, and velum, as well as bony structures such as the plate, mandible, and hyoid^1,2^. The complexity and sophistication of human speech production poses a multitude of open research questions with implications for linguistics and speech science, as well as clinical and technological applications, creating a demand for improved methods for observing and measuring the vocal instrument in action^3,4^.

Real-Time Magnetic Resonance Imaging (RT-MRI) with concurrent audio recording has emerged as an imaging modality that can provide new insights into speech production with all its inherent systematicities and variation between languages, contexts, and individuals^1,3,5,6^. This technique has the unique advantage of monitoring the complete vocal tract safely and non-invasively at relatively high spatial and temporal resolution. Applications of RT-MRI span multiple realms of research including study of: (i) phonetic and phonological phenomena, (ii) spoken language acquisition and breakdown, including the assessment and remediation of speech disorders^1,3^; (iii) dynamics of vocal tract shaping during communicative speech or vocal performance; (iv) articulatory modeling and motor control; (v) speech synthesis and recognition technologies, and (vi) speaker modeling and biometrics.

Several examples of recently published MRI data repositories^7–11^ have demonstrated the value of publicly available datasets to address a multitude of open challenges not only in scholarly research but also in translation into clinical and scientific applications. Open raw datasets have been used to validate and refine advanced algorithms and to train, validate, and benchmark promising recent applications of ideas inspired by artificial intelligence/machine learning methods^12–15^. They are also valued by commercial vendors to showcase performance and generalizability of reconstruction methods. Despite a recognized impact in, for example, the domains of musculoskeletal and brain MRI, there are currently no raw MRI datasets involving the moving vocal tract. Previously published speech MRI datasets^16–20^ (see Table 1) have included reconstructed and processed images for a relatively small number of subjects. In contrast, this dataset provides a larger and more diverse set of speakers, and provides the “raw data” measured by the MRI scanner, which is valuable in that it enables RT-MRI image reconstruction and artifact correction research.

**Table 1:** Comparison of existing speech MRI datasets involving moving vocal tract.

Dataset	The number of speakers	Language studied	Type of imaging data provided	The availability of raw data
Narayanan, S. *et al*.^16^	10 (5 f, 5 m)	American English	RT-MRI with synchronized audio Electromagnetic articulography	No
Kim, J. *et al*.^17^	10 (5 f, 5 m)	American English	RT-MRI with synchronized audio	No
Töger, J. *et al*.^18^	8 (4 f, 4 m)	American English	RT-MRI with synchronized audio Static T2w MRI	No
Sorensen, T. *et al*.^19^	17 (9 f, 8 m)	American English	RT-MRI with synchronized audio 3D volumetric MRI	No
Douros, I. *et al*.^20^	2 (2 m)	French	RT-MRI with synchronized audio 3D volumetric MRI	No
This dataset^52^	75 (40 f, 35 m)	American English	RT-MRI with synchronized audio 3D volumetric MRI Static T2w MRI	Yes

The vocal tract contains multiple rapidly moving articulators, which can change position significantly on a millisecond timescale – an imaging challenge necessitating high temporal resolution adequate for observing these dynamic speech process^6^. While under-sampling of MRI measurements on time-efficient trajectories enables the desired resolution, such measurements are hampered by prolonged computation time for advanced image reconstruction, low signal-to-noise-ratio, and artifacts due to under-sampling and/or rapid differences of magnetic susceptibility^21–23^ at the articulator boundaries, which are of utmost interest in characterizing speech production. These limitations often render present day RT-MRI’s operating point beneath application demands and can introduce bias and increased variance during data analysis. Thus, there is much room for improvements in the imaging technology pipeline. Despite the promise of deep learning/machine learning approaches to provide superior performance both in reconstruction time and image quality, their application to dynamic imaging of fast aperiodic motion with high spatiotemporal resolution and low latency is still in its infancy. We speculate that this is in large measure due to the lack of large-scale public MRI datasets – the cornerstone of machine learning.

This paper presents a unique dataset that offers videos of the entire vocal tract in action, with synchronized audio, imaged along the sagittal plane from 75 participants while they performed a variety of speech tasks. The dataset also includes 3D volumetric vocal tract MRI during sustained speech sounds and high resolution static anatomical T2-weighted upper airway MRI for each participant. Unlike other open datasets for dynamic speech RT-MRI^16–19,24^, the present dataset includes raw MRI data. The raw data is acquired using multiple receiver coils simultaneously and with non-Cartesian, spiral sampling trajectory.

The inclusion of raw dataset can be used to aid the development of algorithms that monitor fast aperiodic dynamics of speech articulators at high spatiotemporal resolution while offering simultaneous suppression of noise and artifacts due to sub-Nyquist sampling^25–28^ or susceptibility^22,23,29–32^. The inclusion of an unprecedented number of 75 speakers can also help improve our scientific understanding of how vocal tract morphology and speech articulation interact and shed light on the stable and variable aspects of speech signal properties across speakers, providing for improved models of speech production in linguistics and speech science research.

In summary, it is our hope and anticipation that the public and free provision of this rich dataset can further foster and stimulate research and innovation in the science of human speech production and its imaging.

## Methods

### Participants

Seventy-five healthy participants (40 females and 35 males; 49 native and 26 non-native American English speakers; age 18–59 years) were included in this study. Each participant filled out a questionnaire on basic demographic information including birthplace, cities raised and lived, and first and second languages. Demographics are summarized in Online-only Table 1. All participants had normal speech, hearing, and reading abilities, and reported no known physical or neurological abnormalities. All participants were cognizant of the nature of the study, provided written informed consent, and were scanned under a protocol approved by the Institutional Review Board of the University of Southern California (USC). The data were collected at the Los Angeles County – USC Medical Center between January 24, 2016 and February 24, 2019.

### Experimental overview

**Fig. 1:**
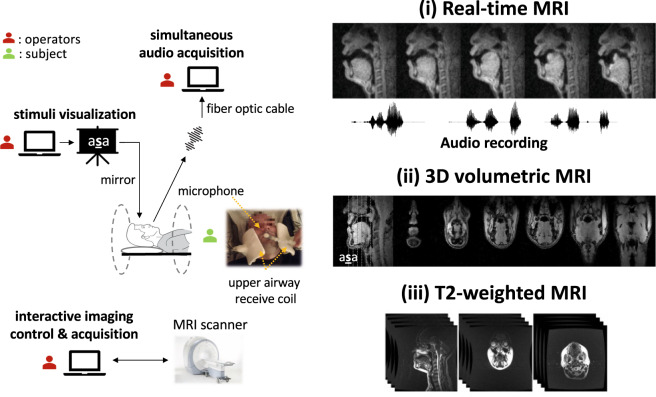
Data acquisition workflow and data records. (Left) Data were acquired at 1.5 Tesla using the custom upper-airway coil located in close proximity to the participant’s upper airway structures. The participant visualized the stimuli through a mirror-projector setup and audio was recorded through an MR-compatible microphone simultaneous with the RT-MRI. The scanner operator used a custom interactive imaging interface with the scanner hardware to control and acquire the data for the RT-MRI session. (Right) The recorded MRI data were: (i) dynamic, 2D real-time MRI of the vocal tract’s mid-sagittal slice at 83 frames per second during production of a comprehensive set of scripted and spontaneous speech material, along with synchronized audio recording; (ii) static, 3D volumetric images of the vocal tract, captured during sustained production of sounds or postures, 7 seconds each; (iii) T2-weighted volumetric images at rest position, capturing fine detail anatomical characteristics of the vocal tract.

Three types of MRI data were recorded for each participant: (i) dynamic, real-time MRI of the vocal tract’s mid-sagittal slice at 83 frames per second during production of a comprehensive set of scripted and spontaneous speech material, averaging 17 minutes per participant, along with synchronized audio; (ii) static, 3D volumetric images of the vocal tract, captured during sustained production of sounds from the full set of American English vowels and continuant consonants, 7 seconds each; (iii) T2-weighted volumetric images at rest position, capturing fine detail anatomical characteristics of the vocal tract (See Fig. 1).

All data were collected using a commercial 1.5 Tesla MRI scanner (Signa Excite, GE Healthcare, Waukesha, WI) with gradients capable of 40 mT/m amplitude and 150 mT/m/ms slew rate. A custom 8‐channel upper airway receiver coil array^26^, with four elements on each side of the participant’s cheeks, was used for signal reception. Compared to commercially available coils that are designed for neurovascular or carotid artery imaging, this custom coil has been shown to provide 2-fold to 6-fold higher signal-to-noise-ratio (SNR) efficiency in upper airway vocal tract regions of interests including tongue, lips, velum, epiglottis, and glottis. The participants, while imaged, were presented with scripts of the experimental stimuli via a projector-mirror setup^33^. Acoustic audio data were recorded inside the scanner using commercial fiber-optic microphones (Optoacoustics Inc., Yehuda, Israel) concurrently with the RT-MRI data acquisition using a custom recording setup^34^.

### Stimuli and linguistic justification

The stimuli were designed to efficiently capture salient, static and dynamic, and articulatory and morphological aspects of speech production of American English in a single 90-minute scan session. Table 2 lists the speech stimuli used for the RT-MRI data collection of the first 45-minute sub scan session. Each individual task was designed to be performed within 30 seconds at a normal speaking rate, however the actual recordings varied in duration depending on the length of the task and the natural speaking rate of the individual. The stimulus set contained material to elicit both scripted speech and spontaneous speech. The scripted speech tasks were consonant production in symmetric vowel-consonant-vowel context, vowels /V/ produced between the consonants /b/ and /t/, i.e., in /bVt/ contexts, four phonologically rich sentences^35^, and three reading passages commonly used in speech evaluation and linguistic studies^36–38^. Scripted instructions to produce several gestures were also included. These gestures were clenching, wide opening of mouth, yawning, swallowing, slow production of the sequence /i/-/a/-/u/-/i/, tracing of the palate with the tongue tip, and singing “la” at their highest and lowest pitches. The stimuli in the scripted speech were repeated twice. The spontaneous speech tasks were to describe the content and context of five photographs (Supplemental Fig. 1) and to answer five open-ended questions (e.g., “What is your favorite music?”). The full scripts and questions are provided in Table 3.

**Table 2:** Speech experiment stimuli for 2D RT-MRI.

Category	Stimulus Index	Stimulus Name	Description	Duration (sec)
Scripted speech	01, 02, 03	vcv[1–3]	Consonants in symmetric /VCV/	30 (x3)
	04	bvt	Vowels in /bVt/	30
	05	shibboleth	Four phonologically rich sentences^34^	30
	06	rainbow	Rainbow passage^35^	30
	07, 08	grandfather[1–2]	Grandfather passage^36^	30 (x2)
	09, 10	northwind[1–2]	Northwind and the sun passage^37^	30 (x2)
	11	postures	Postures	30
	Repetition of the above scripted speech tasks			30 (x11)
Spontaneous speech	12–16	picture[1–5]	Description of pictures	30 (x5)
	17–21	topic[1–5]	Discussion about topics	30 (x5)

**Table 3:** Full scripts and questions of speech experiment stimuli for 2D RT-MRI.

Stimulus Index	Script or question/topic
vcv1	apa upu ipi ata utu iti aka uku iki aba ubu ibi ada udu idi aga ugu igi
vcv2	atha uthu ithi asa usu isi asha ushu ishi ama umu imi ana unu ini ala ulu ili
vcv3	afa ufu ifi ava uvu ivi ara uru iri aha uhu ihi awa uwu iwi aya uyu iyi
bvt	beet bit bait bet bat pot but bought boat boot put bite beaut bird boyd abbot
shibboleth	She had your dark suit in greasy wash water all year. Don’t ask me to carry an oily rag like that. The girl was thirsty and drank some juice followed by a coke. Your good pants look great however your ripped pants look like a cheap version of a k-mart special is that an oil stain on them.
rainbow	When the sunlight strikes raindrops on the air they act as a prism and form a rainbow. The rainbow is a division of white light into many beautiful colors. These take the shape of a long round arc with its path high above and its two ends apparently beyond the horizon. There is according to legend a boiling pot of gold at one end. People look but no one ever finds it. When a man looks for something beyond his reach his friends say he is looking for the pot of gold at the end of the rainbow.
grandfather1	You wish to know all about my grandfather. Well, he is nearly ninety three years old yet he still thinks as swiftly as ever. He dresses himself in an old black frock coat usually several buttons missing. A long beard clings to his chin, giving those who observe him a pronounced feeling of the utmost respect. When he speaks, his voice is just a bit cracked and quivers a bit.
grandfather2	Twice each day he plays skillfully and with zest upon a small organ. Except in the winter when the snow or ice prevents, he slowly takes a short walk in the open air each day. We have often urged him to walk more and smoke less but he always answers, “banana oil” grandfather likes to be modern in his language.
northwind1	The north wind and sun were disputing which was the stronger, when a traveler came along wrapped in a warm cloak. They agreed that the one who first succeeded in making the traveler take off his cloak should be considered stronger than the other.
northwind2	Then the north wind blew as hard as he could, but the more he blew the more closely did the traveler fold his cloak around him and at last the north wind gave up the attempt. Then the sun shone out warmly and immediately the traveler took off his cloak and so the north wind was obliged to confess that the sun was stronger of the two.
postures	clench, open wide & yawn, swallow, eee … aahh … uuww … eee, trace palate with tongue tip, Sing “la” at your highest note, Sing “la” at your lowest note
topic1	My favorite music
topic2	How do I like LA?
topic3	My favorite movie
topic4	Best place I’ve been to
topic5	My favorite restaurant

Table 4 lists the speech stimuli used for the 3D volumetric data collection of a 30-minute part of the scan session. Each individual task was designed for a participant to sustain vowels, consonant sounds, or to maintain postures for 7 seconds. The stimulus set contained consonant production in symmetric vowel-consonant-vowel context, vowels /V/ produced between the consonants /b/ (or /p/) and /t/, and production of several postures. All of the stimuli were repeated twice.

**Table 4:** Speech experiment stimuli for 3D volumetric static MRI.

Category	Stimulus Index	Stimulus Name	Description	Duration (sec)
Vowels in /bVt/			Sustain sound at vowel	7 (x13)
	01	beet	beet	
	02	bit	bit	
	03	bait	bait	
	04	bet	bet	
	05	bat	bat	
	06	pot	pot	
	07	but	but	
	08	bought	bought	
	09	boat	boat	
	10	boot	boot	
	11	put	put	
	12	bird	bird	
	13	abbot	abbot	
Consonants in symmetric /VCV/			Sustain sound at consonant	7 (x14)
	14	afa	afa as in food	
	15	ava	ava as in voice	
	16	atha_thing	atha as in thing	
	17	atha_this	atha as in this	
	18	asa	asa as in soap	
	19	aza	aza as in zipper	
	20	asha	asha as in shoe	
	21	agea	agea as in beige	
	22	aha	aha as in happy	
	23	ama	ama as in rim	
	24	ana	ana as in pin	
	25	anga	anga as in ring	
	26	ala	ala as in late	
	27	ara	ara as in rope	
Postures	28	breathe	Breathe normally with your mouth closed, resting.	7 (x6)
	29	clench	Clench your teeth and hold.	
	30	tongue	Stick your tongue out as far as you can,	
	31	yawn	Pull back your tongue as far into the mouth as you can, and hold (like yawning).	
	32	tip	Raise the tip of your tongue to the middle of your palate, and hold, and hold.	
	33	hold	Hold your breath.	
Repetition of the above tasks				7 (x33)

### RT-MRI acquisition

RT-MRI acquisition was performed using a 13-interleaf spiral-out spoiled gradient-echo pulse sequence^33^. This is an efficient scheme for sampling MR measurements, each with the different initial angle being interleaved by the bit-reversed order in time^39^. The 13 spiral interleaves, when collected together, fulfil the Nyquist sampling rate. Imaging parameters were: repetition time = 6.004 ms, echo time = 0.8 ms, field-of-view (FOV) = 200 × 200 mm^2^, slice thickness = 6 mm, spatial resolution = 2.4 × 2.4 mm^2^ (84 × 84 pixels), receiver bandwidth = ± 125 kHz, flip angle = 15°. Imaging was performed in the mid-sagittal plane, which was prescribed using a real‐time interactive imaging platform^40^ (RT-Hawk, Heart Vista, Los Altos, CA). Real-time visualization was implemented within the custom platform by using a sliding window gridding reconstruction^41^ to ensure participant’s compliance with stimuli and to detect substantial head movement.

Acquisition was divided into 20–40 second task intervals presented in Table 2, each followed by a pause of the same duration so as to allow enough a brief break for the participant prior to the next task and to avoid gradient heating.

### RT-MRI Reconstruction

The dataset provides one specific image reconstruction that has been widely used in speech production research^26,33^. This image reconstruction involves optimizing the following cost function:

1 $${{\rm{\min }}}_{m}{\left\Vert Am-d\right\Vert }_{2}^{2}+\lambda {\left\Vert {\nabla }_{t}m\right\Vert }_{1}$$

where *A* represents the encoding matrix that models for the non-uniform fast Fourier transform and the coil sensitivity encoding, *m* is the dynamic image time series to be reconstructed, *d* is the acquired multiple-coil raw data, λ is the regularization parameter, ∇_*t*_ is the temporal finite difference operator, and ${\parallel \,\cdot \,\parallel }_{2}^{2}$ and ${\parallel \cdot \parallel }_{1}$ are *l*_2_ and *l*_1_ norms, respectively. The coil sensitivity maps were estimated using the Walsh method^42^ from temporally combined coil images. The regularization term encourages voxels to be piecewise constant along time. This regularization has been successfully applied to speech RT-MRI^26,43,44^ and a variety of other dynamic MRI applications where the primary features of interest are moving tissue boundaries^45–47^.

Our reference implementation solves Equation [1] using nonlinear conjugate gradient algorithm with Fletcher-Reeves updates and backtracking line search^48^. The algorithm was terminated either at 150 maximum iterations or the step size fell below <1e-5 during line search. Reference images were reconstructed using 2 spiral arms per time frame, resulting in a temporal resolution of 83.28 frames per second. This temporal resolution is enough to capture important articulator motions^1^, but reconstruction at different temporal resolutions is also possible with the provided dataset by adjusting the number of spiral arms per time frame. The algorithm was implemented in both MATLAB (The MathWorks, Inc., Natick, MA) and Python (Python Software Foundation, https://www.python.org/). The provided image reconstruction was performed in MATLAB 2019b on a Xeon E5-2640 v4 2.4 GHz CPU (Intel, Santa Clara, CA) and a Tesla P100 GPU (Nvidia, Santa Clara, CA). Reconstruction time was 160.69 ± 1.56 ms per frame. Parameter selection of *λ* is described in the technical validation section below.

### Audio data

One main microphone was positioned ~0.5 inch away from the participant’s mouth. The microphone signal was sampled at 100 kHz each. The data were recorded on a laptop computer using a National Instruments NI-DAQ 6036E PCMCIA card. The audio sample clock was hardware-synchronized to the MRI scanner’s 10 MHz master clock. The audio recording was started and stopped using the trigger pulse signal from the scanner. The real-time audio data acquisition routine was written in MATLAB. Audio was first low-pass filtered and decimated to a sampling frequency of 20 kHz. The recorded audio was then enhanced using a normalized least-mean-square noise cancellation method^34^ and was aligned with the reconstructed MRI video sequence to aid linguistic analysis.

### 3D Volumetric MRI of sustained sounds

An accelerated 3D gradient-echo sequence with Cartesian sparse sampling was implemented to provide static high-resolution images of the full vocal tract during sustained sounds or postures. Imaging parameters were: repetition time = 3.8 ms, spatial resolution = 1.5625 × 1.5625 × 1.5625 mm^3^, FOV = 250 × 250 × 125 mm^3^ (respectively in the anterior-posterior, superior-inferior, and left-right directions), image matrix size = 160 × 160 × 80, and flip angle = 5°. The central portion (40 × 20) of the k_y_-k_z_ space was fully sampled to estimate the coil sensitivities from the data itself. The outer portion of the k_y_-k_z_ space was sampled using a sparse Poisson-Disc sampling pattern, which together resulted in 7-fold net acceleration, and a total scan time of 7 seconds.

Data were acquired while the participants sustained for 7 seconds a sound from the full set of American English vowels and continuant consonants (Table 4). The stimuli were presented to the participant via a projector-mirror setup, and upon hearing a “GO” signal given by the scanner operator, a participant started to sustain the sound or posture; this was followed by the operator triggering the acquisition manually. Participants sustained the postures as long as they could hear the scanner operating. A recovery time of 5–10 seconds was given to the participant between the stimuli.

Image reconstruction was performed off-line by a sparse-SENSE constrained reconstruction^49^. This method reconstructs images from multichannel, undersampled data, similar to the optimization problem shown in Equation [1]. One difference is that the non-Cartesian Fourier encoding in matrix *A* is replaced by undersampled Cartesian Fourier encoding. Also the temporal finite difference sparsity constraint is replaced by an isotropic spatial total variation^33,50^. The reconstruction was achieved using the open-source Berkeley Advanced Reconstruction Toolbox (BART)^51^; the image reconstruction was performed in MATLAB using GPU acceleration.

### T2-Weighted MRI at rest

Fast spin echo-based sequence was performed to provide high-resolution T2-weighted images with fine detail anatomical characteristics of the vocal tract at rest position. Imaging was run to obtain full sweeps of the vocal tract in the axial, coronal, and sagittal orientations, for each of which the number of slices ranges from 29 to 70, depending on the size of the vocal tract. Imaging parameters were: repetition time = 4600 ms, echo time = 120–122 ms, slice thickness = 3 mm, in-plane FOV = 300 × 300 mm^2^, in-plane spatial resolution = 0.5859 × 0.5859 mm^2^, number of averages = 1, echo train length = 25, and scan time = approximately 3.5 minutes per orientation.

## Data Records

This dataset is publicly available in figshare^52^. The total size of this dataset is approximately 966 GB. It contains (i) raw 2D sagittal-view RT-MRI data, reconstructed images and videos, and synchronized denoised audio, (ii) 3D volumetric MRI images, and (iii) T2-weighted MRI images. The data for participant (subject) XYZ is contained in folder with identifier subXYZ (e.g., *sub001/)* and organized into three main folders: 2D RT-MRI data are located in the subfolder *2drt/* (e.g., *sub001/2drt/*), 3D volumetric images in *3d/*, and T2-weighted images in *t2w/*. The contents and data structures of the dataset are detailed as follows.

### RT-MRI

Raw RT-MRI data are provided in the vendor-agnostic MRD format (previously known as ISMRMRD, https://ismrmrd.github.io/)^53^, which stores k-space MRI measurements, k-space location tables, and sampling density compensation weights. Parameters for the acquisition sequence are contained in the file header information. In addition, this dataset includes reconstructed image data for each participant and task in HDF5 format, audio files in WAV format, and videos in MPEG-4 format.

For each participant, RT-MRI raw data is contained in the subfolder *raw/ (*e.g., *sub001/2drt/raw/*), reconstructed image data in *recon/*, co-recorded audio (after noise cancellation) in *audio/*, and reconstructed speech videos with aligned audio in *video/*. Table 5 summarizes the data structure and naming conventions for this dataset.

**Table 5:** Naming of folders and files.

Category	Data folder	Filename convention	Description
RT-MRI	subXYZ/2drt/raw/	*<subject-identifier>*_2drt_<*stimulus-index>_<stimulus-name>*_r<*repetition>*_raw.h5 (e.g., sub001_2drt_01_vcv1_r1_raw.h5)	Raw RT-MRI k-space data in MRD format
	subXYZ/2drt/recon/	*<subject-identifier>*_2drt_<*stimulus-index>_<stimulus-name>*_r<*repetition>*_recon.h5	Reconstructed RT-MRI image data in HDF5 format
	subXYZ/2drt/audio/	*<subject-identifier>*_2drt_<*stimulus-index>_<stimulus-name>*_r<*repetition>*_audio.wav	Co-recorded audio data in WAV format
	subXYZ/2drt/video/	*<subject-identifier>*_2drt_<*stimulus-index>_<stimulus-name>*_r<*repetition>*_video.mp4	Videos of speech task with aligned audio in MPEG-4 format
3D volumetric	subXYZ/3d/recon/	*<subject-identifier>*_3d_<*stimulus-index>_<stimulus-name>*_r<*repetition>*_recon.mat (e.g., sub001_3d_13_abbot_r1_recon.mat)	Reconstructed volumetric image data in MAT format
	subXYZ/3d/snapshot/	*<subject-identifier>*_3d_<*stimulus-index>_<stimulus-name>*_r<*repetition>*_snapshot.png	Mid-sagittal slice image in PNG format
T2-weighted	subXYZ/t2w/dicom/	*<orientation-index><slice-index>*.dcm (e.g., 00010001.dcm)	T2-weighted image in DICOM format

Participant (Subject) identifiers correspond to Online-only Table 1, column *Subject ID*. Stimulus indices correspond to Tables 2 and 4, column *Stimulus Index*. Stimulus names correspond to Tables 2 and 4, column *Stimulus Name*.

### 3D Volumetric MRI of sustained sounds

3D volumetric MRI data contains reconstructed image data and imaging parameters in MAT format (MATLAB, The MathWorks, Inc., Natick, MA) in the subfolder *recon/ (*e.g., *sub001/3d/recon/)* and a mid-sagittal slice image in PNG format in the subfolder *snapshot/*.

### T2-Weighted MRI at rest

T2-weighted image data from axial, coronal, and sagittal orientations are stored in Digital Imaging and Communications in Medicine (DICOM) format in the subfolder *dicom/ (*e.g., *sub001/t2w/dicom/)*.

The imaging DICOM files were de-identified using the Clinical Trial Processor (CTP) developed by the Radiological Society of North America (RSNA)^54^. Specifically, data anonymization was completed using a command line tool developed in the Java programming language (http://mircwiki.rsna.org/index.php?title=The_DicomAnonymizerTool). All images followed the standardized DICOM format and some of the attributes were removed or modified to preserve privacy of the participants, specifically: *PatientName* was modified to follow the *subXYZ* pattern, and the original study dates were shifted by the same offset for all participants.

### Metadata

We provide presentation slides that contain the experimental stimuli including scripts and pictures used for the visualization to the participants, in PPT format in *Stimuli.ppt*. Demographic information for each participant is contained in XLSX format in *Subjects.xlsx*. This meta file contains sex, race, age (at the time of scan), height (cm), weight (kg), origin, birthplace, cities raised and lived, L1 (first language), L2 (second language, if any), L3 (third language, if any), and the first language and birthplace of each participant’s parents.

Additionally, meta information for each participant and *RT-MRI* task is contained in JSON format in *metafile_public_* < *timestamp* > *.json*. For each participant, we include the following demographic information: 1) L1 (first language), 2) L2 (second language, if any), 3) sex (M/F), 4) age (years at the time of scan). Further, visual and audio quality assessment scores are provided using a 5-level Likert scale for each participant stratified into categories: off-resonance blurring (1, very severe; 2, severe; 3, moderate; 4, mild; 5, none), video SNR (1, poor; 2, fair; 3, good; 4, very good; 5, excellent), aliasing artifacts (1, very severe; 2, severe; 3, moderate; 4, mild; 5, none), and audio SNR (1, poor; 2, fair; 3, good; 4, very good; 5, excellent). Specifically, an MRI expert with 6 years of experience in reconstructing and reading speech RT-MRI examined all the RT-MRI videos reconstructed for each participant and assessed and scored their visual and audio quality. For each task of the individual participants, the information about task’s index, name, existence of file, and notes taken during data inspection by inspectors are also contained in the meta file.

## Technical Validation

### Data inspection

Four inspectors manually performed qualitative data inspection for the datasets. After reconstruction, all images were converted from HDF5 format to MPEG-4 format, at which time co-recorded audio (WAV format) was integrated. All qualitative data inspection was performed manually based on MPEG-4 format videos. Included in the dataset were files that met all of the following criteria:

1. The video (MPEG-4) exists (no data handling failure).
2. The audio recording (WAV) exists (no data handling failure).
3. The video and audio are synchronized (based on inspection by a human).

After the inspection, 75 participants were included in this dataset. It should be noted that although three participants (sub18, sub74, and sub75) present severe radio-frequency-interference artifacts that were later determined to be from a leak in the MRI radio-frequency cage, we included those three participants in this dataset in the hopes of potentially facilitating development of a digital radio-frequency interference correction method. Files that did not exist (criteria 1) were annotated for each task and participant in the meta file (i.e., *metafile_public_* < *timestamp* > *.json*).

**Fig. 2:**
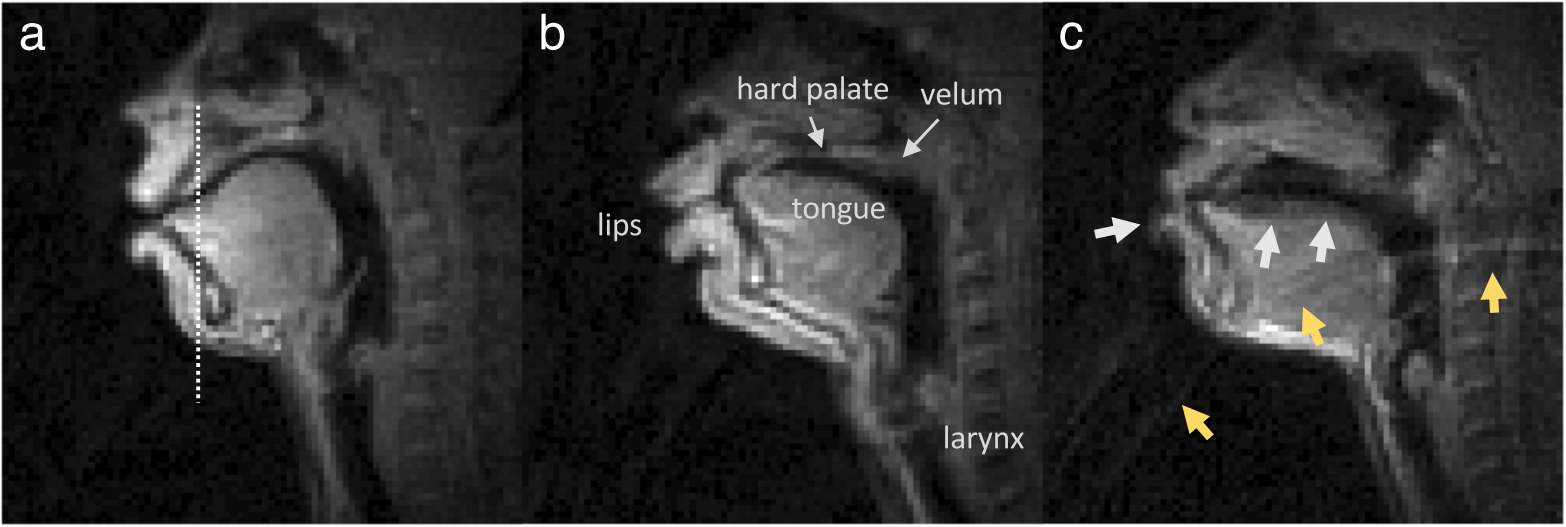
Typical data quality of 2D real-time speech imaging, shown in mid-sagittal image frames from three example participants: (**a**) sub35 (male, 21 yrs, native American English speaker), (**b**) sub51 (male, 33 yrs, non-native speaker), (**c**) sub58 (female, 28 yrs, non-native speaker). The mid-sagittal image frames depict the event of articulating the fricative consonant [*θ*] in the word “uthu” (stimulus “vcv2”), where the tongue tip contacts the upper teeth. (**a**) and (**b**) are considered to have very high quality, based on high SNR and no noticeable artifact. (**c**) is considered to have moderate quality, based on good SNR and mild image artifacts; the white arrows point to blurring artifacts due to off-resonance while the yellow arrows point to ringing artifacts due to aliasing.

Figure 2 shows representative examples of the data quality from three participants that are included in this dataset (sub35, sub51, and sub58). Note that all 75 participants exhibited acceptable visualization of all soft tissue articulators. However, two types of artifacts were commonly observed. 1) *Blurring artifact due to off-resonance (white arrows*, Fig. 2c*)*: This artifact appears as blurring or signal loss predominantly adjacent to air-tissue boundaries that surround soft tissue articulators. It is induced in spiral imaging by rapid changes of local magnetic susceptibility between the air and tissue. This artifact correction is still an active research area, including methods for a simple zeroth order frequency demodulation to advanced model-based^21,22,30^ and data-driven approaches^23,55^. At present, we perform zeroth order correction during data acquisition as part of our routine protocol. 2) *Ringing artifact due to aliasing (yellow arrows*, Fig. 2c*)*: This artifact appears as an arc-like pattern centered at the bottom-right corner outside the FOV. It is caused by a combination of gradient non-linearity and non-ideal readout low-pass filter when a strong signal source appears outside the unaliasing FOV. This artifact does not overlap with the articulator surfaces that are most important in the study of speech production. However, avoiding and/or correcting this artifact would improve overall image quality and potentially enable the use of a smaller FOV.

**Fig. 3:**
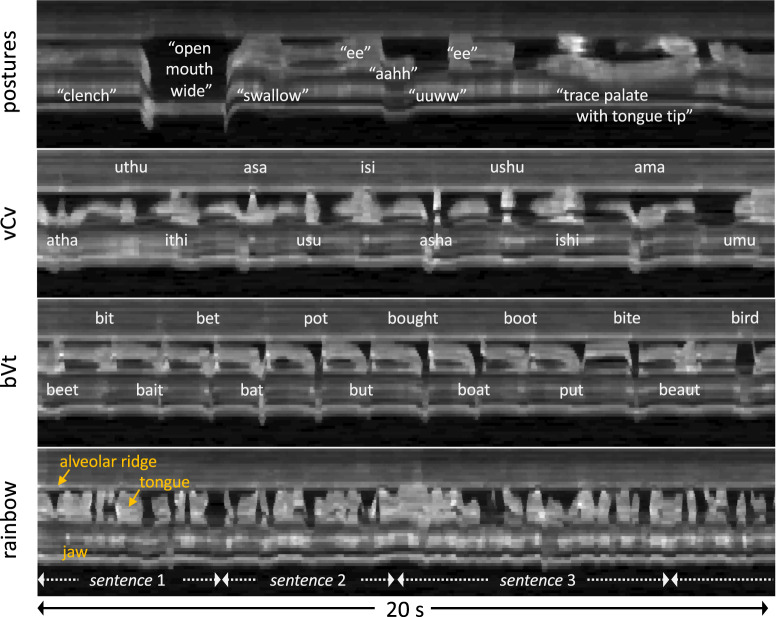
Image vs. time profiles during the first 20 seconds of four different stimuli for sub35. Profiles show the time evolution of the cut depicted by the dotted line in the image frame shown in Fig. 2a (sub35). The rows visualize different examples of stimuli: “postures,” “vcv2,” “bvt,” and “rainbow” passage. The set of stimuli covers a wide range of articulator postures and tongue velocities.

**Fig. 4:**
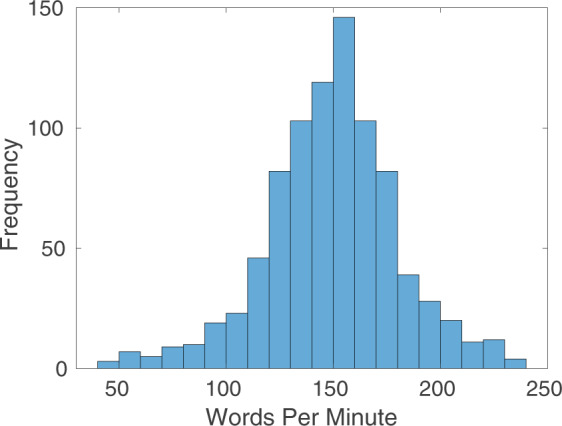
Histogram of words per minute during scripted speech stimuli including “shibboleth,” “rainbow,” “grandfather[1–2],” and “northwind[1–2]”.

Figure 3 shows representative examples of the diverse speech stimuli that are included in this dataset. The intensity vs. time profiles visualize the first 20 seconds of four representative stimuli from sub35, shown in Fig. 2a. These examples show the variety of patterns and speed of movement of the soft tissue articulators observed within a speaker as a function of the speech stimuli performed. Figure 4 contains a histogram of the speaking rate in read sentences^56^. The overall mean speaking rate was 149.2 ± 31.2 words per minute. The statistics are calculated from the read “shibboleth,” “rainbow,” “grandfather[1–2],” and “northwind[1–2]” stimuli for all speakers. These selected stimuli are composed of read full sentences.

**Fig. 5:**
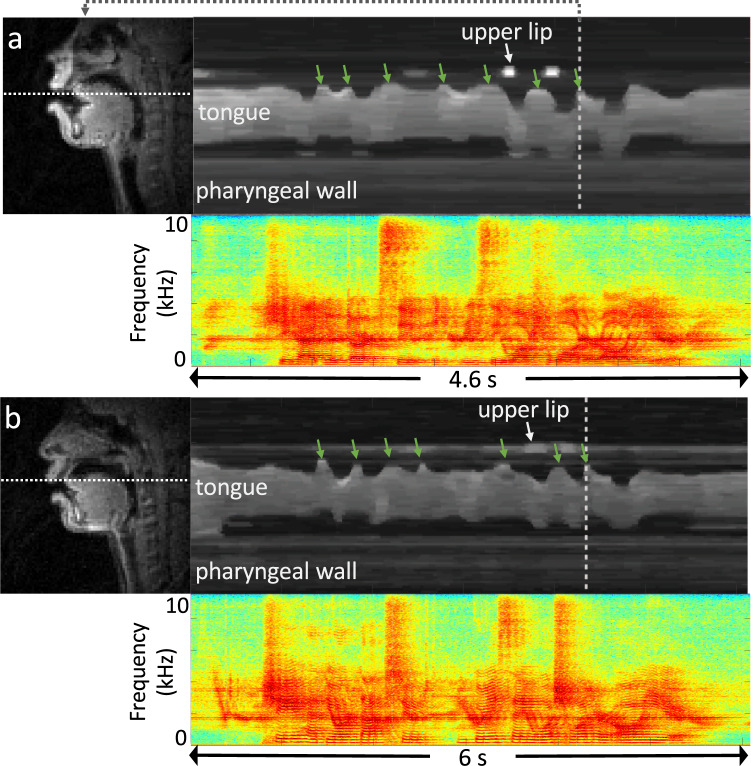
Variability in the articulation of the same sentence between two speakers: (**a**) sub35, (**b**) sub41. The time profile and audio spectrum correspond to the first sentence “She had your dark suit in greasy wash water all year” in the stimulus of “shibboleth” from each speaker. The green arrows point to several noticeable time points at which the tongue tip contacts the upper teeth/alveolar ridge.

Figure 5 illustrates variability within the same speech stimuli across different speakers. The image time profiles correspond to the period of producing the first sentence “She had your dark suit in greasy wash water all year” in the stimulus “shibboleth” from sub35 and sub41. Although both speakers share the critical articulatory events (see green arrows), the timing and pattern vary depending on the speakers.

### Regularization parameter selection for image reconstruction for RT-MRI

**Fig. 6:**
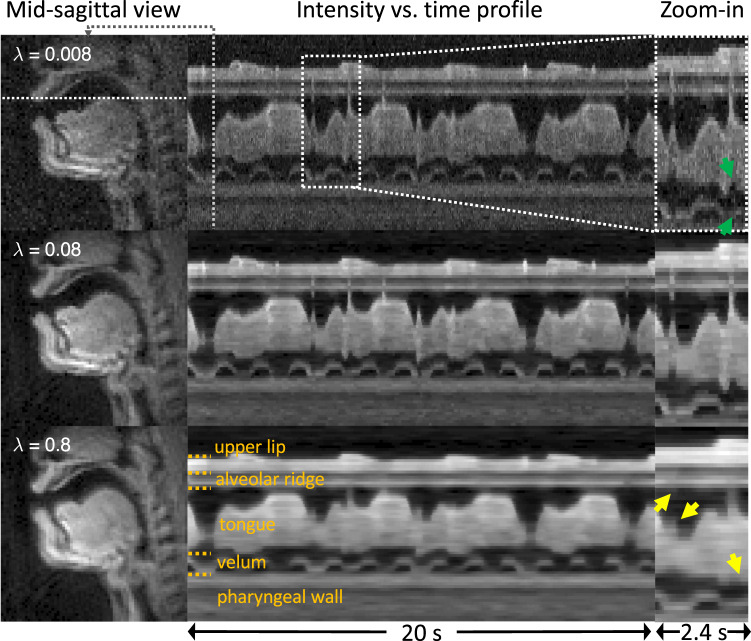
Illustration of the impact of reconstruction parameter λ on image quality. Data are from sub15 (male, 26 yrs, native American English speaker). (Left) Mid-sagittal image frames during speaking. (Middle) The intensity vs. time profiles for stimulus “vcv1.” (Right) Zoomed-in time profiles. Different rows correspond to different λ values. For a smaller λ (=0.008 C), the reconstruction shows a higher noise level and obscured articulatory event (green arrows), whereas for a higher λ (=0.8 C), the noise level decreases but the temporal smoothing artifact is evident in regions indicated by yellow arrows. λ = 0.08 C yields an acceptable noise level while showing adequate temporal fidelity and therefore was selected for the optimal value for the reconstruction.

We performed a parameter sweep and qualitative evaluation on a subset of the data to select a regularization parameter for the provided reconstructions. Ten stimuli from ten different participants were randomly selected. The regularization parameter λ was swept in the range between 0.008 C and 1 C in a logarithmic scale. Here, C represents the maximum intensity of the zero-filled reconstruction of the acquired data. Figure 6 shows a representative example of the impact of λ on the reconstructed image quality. A small λ (=0.008 C) exhibits high noise level in the reconstruction (top, Fig. 6). A higher λ (=0.8 C) reduces the noise level but exhibits unrealistic temporal smoothing as shown in the intensity vs. time profiles (yellow arrows, Fig. 6). The optimal parameter 0.08 C was selected by consensus among four experts in the area of MRI image reconstruction and/or speech production RT-MRI. Once the λ was optimized for the subset of the data, reconstruction was performed for all datasets. We have empirically observed that the choice of λ appears robust across all of the datasets.

## Usage Notes

Several papers have been published by our group in which methods are directly applied to a subset of this dataset for the reconstruction and artifact correction of the RT-MRI data. These include auto-calibrated off-resonance correction^22^, deblurring using convolutional neural networks^23,57^, aliasing artifact mitigation^58^ (the artifact marked by the yellow arrows in Fig. 2c), and super-resolution reconstruction^59^.

Additionally, several tools have been developed at our site for the analysis and modelling of reconstructed real-time MRI data. These include a graphical user interface for efficient visual inspection^16^ and implementations of grid-based tracking of air-tissue boundaries^60–62^, region segmentation and factor analysis^63–65^, neural network-based edge detection^66,67^; region of interest (ROI) analysis^68–70^ and centroid tracking^71^. Some of these tools are also made available; see code repository at https://github.com/usc-mrel/usc_speech_mri.git as well as Ramanarayanan *et al*.^3^, Hagedorn *et al*.^4^, and Toutios *et al*.^24,72^ for detailed reviews.

Our group has developed data-driven computational models including an anatomically-guided factor analysis approach of vocal tract contours derived automatically from RT-MRI to provide a compact representation of vocal tract shapes allowing utterances to be decomposed into a linear combination of time functions for the weights of linguistically interpretable vocal-tract deformations^64^. We also developed the Convex Hull Convolutive Non-Negative Matrix Factorization approach to learn and decompose temporal patterns in multivariate time-series data such as vocal tract constriction trajectories^73^. We have deployed computational modeling in specific studies including: (i) Development of a framework for determining the test-retest repeatability of quantitative speech biomarkers across experimental conditions and tasks, and sharing test-retest RT-MRI data freely for research use^18^; (ii) Investigation of synergies for different constriction tasks in VCV sequences with a RT-MRI biomarker computed using a statistical model of vocal tract forward kinematics^65^, supporting the claim that inter-articulator coordination differs depending on the active synergies; (iii) Development of a new vocal tract length estimation model^74^ that affords normalization of interspeaker variability and facilitates acoustic comparisons across speakers; (iv) Investigation of speed-accuracy tradeoffs (Fitts’ law) across syllable position and subjects, which led to a theoretical account within models of speech motor control^75^; (v) Design of a modular architecture for articulatory synthesis from a gestural specification comprising relatively simple models for the glottis, aero-acoustics, and the vocal tract^76^.

RT-MRI vocal tract imaging data also have facilitated a range of linguistic studies focused on the composition and internal structure of speech sounds such as for example the production of liquid—rhotic and lateral—multi-gesture segments. The studies have probed the articulation of English liquid consonants in syllable onsets and codas, comparing tongue posture produced in syllable margins adjacent to different vowels^77^. Laterals exhibited greater temporal and spatial independence between their tongue tip and tongue rear gestural components, whereas rhotics were produced with a variety of speaker-specific postures but showed less variation across syllable positions than laterals. Harper *et al*.^78^ examined the multiple component gestures of American English/ɹ/—palatal, pharyngeal, and labial—to understand each gesture’s relative contribution to third formant lowering. Data from the USC-TIMIT RT-MRI corpus^16^ were analyzed to determine the location, length, and aperture for each constriction gesture of /ɹ/, with formant values extracted at the time of maximal constriction for each gesture. Results suggest that each gesture’s contribution to F3 depends on between- and within-speaker variation in the articulation of /ɹ/, as variation in the length and location of the palatal and pharyngeal constrictions is associated with differences in their relative effect size on F3 lowering. In another study, Harper *et al*.^79^ observed RT-MRI data for L2 rhotic production for English learners, finding that the component pharyngeal gesture is particularly difficult for L2 learners whose L1 rhotics lack such a constriction, such as French and Greek. Finally, Monteserín *et al*.^80^ compared the articulation of coda rhotic taps and laterals in Puerto Rican Spanish, which often fail to be discriminated by speakers of other Spanish dialects, and found support for the hypothesis that they are produced with distinct articulatory gestures. Oh *et al*.^81,82^ use RT-MRI to examine glottalic consonants in Hausa. These non-pulmonic consonants such as ejectives and implosives are produced by manipulating pressure in the supralaryngeal vocal tract by means of changes in the vertical larynx position—lowering for implosives and raising for ejectives. RT-MRI has for the first time provided quantitative descriptions of these larynx actions and their intergestural timing with their critically coordinated oral constrictions. This study finds that implosives and voiced stops contrast in their internal intergestural timing, not in magnitude of larynx lowering. The observed coordination between vertical larynx movement and the oral closure can be analyzed as in-phase coupling for pulmonic stops but anti-phase (sequential) coupling for glottalic consonants, implying that in addition to the combination of gestures that comprise a segment, internal phasing relations (or timing) of these gestures must also be encoded in the phonological representation of these complex gestural molecules. In a complementary study, Proctor *et al*.^77,83^ use RT-MRI to illuminate the production of click consonants by a speaker of Khoekhoegowab, revealing that different articulatory actions are used to rarefy the mid-oral cavity in different clicks. Dental clicks are produced with tongue body lowering with a laminal tongue front constriction, while alveolar clicks are characterized by a more apical, rapid tongue tip release with the tongue rear remaining raised in the uvular region.

Finally, the RT-MRI methods have also inspired studies focused on the nature of vocal tract shaping in specific clinical contexts. Lander Portnoy, Goldstein, and Narayanan^84^ documented the speech of a patient who has undergone partial glossectomy, at both pre-operative and post-operative time points. They used RT-MRI to observe changes in the variability and principal axes of movement for the tongue. Hagedorn *et al*.^85^ presented an RT-MRI analysis of speech errors in a speaker with acquired apraxia, capturing two types of gestural intrusion errors in a word pair repetition task, as well as intrusion errors in nonrepetitive speech, multiple silent initiation gestures at the onset of speech, and covert (unphonated) articulation of entire monosyllabic words. Lastly, McMicken *et al*.^86^ and Toutios *et al*.^87^ employed RT-MRI to probe the speech production patterns of a speaker with congenital aglossia, a rare syndrome in which an individual is born without a full tongue, who nonetheless has acquired the ability to produce highly intelligible speech. The study found that the speaker, in the absence of a tongue tip, produces plosive consonants that are perceptually similar to /t, d/ by using a bilabial constriction with a significantly increased anteroposterior extent (relative to her /p, b/ productions), accompanied by an increase in closure duration and a widening of the pharynx^87^.

## Supplementary information

Supplemental Figure 1

## Data Availability

This dataset is accompanied by a code repository (https://github.com/usc-mrel/usc_speech_mri.git) that contains examples of software and parameter configurations necessary to load and reconstruct the raw RT-MRI in MRD format. Specifically, the repository contains demonstrations to illustrate and replicate results of Figs. 2–6. Code samples are available in MATLAB and Python programming languages. All software is provided free to use and modify under the MIT license agreement.

## Online-only Table

**Online-only Table 1:** Demographic information of participants; F: Female, M: Male, L1: first language, L2: second language (if any).

Subject ID	Sex	Age at the time of scan (years)	L1	L2	City raised
sub001	F	24	Mandarin	English	Hohhot, China
sub002	F	26	English	—	Phoenix, AZ
sub003	F	19	English	Mandarin	Alhambra, CA
sub004	M	21	English	—	Portland, OR
sub005	F	27	English	Spanish	Ashland, KY
sub006	M	27	English	—	Exton, PA
sub007	F	30	English	Spanish	Alameda, CA
sub008	M	32	Telugu	Hindi	Hyderabad, India
sub009	M	27	English	—	*Unknown*
sub010	F	21	English	—	Cranston, RI
sub011	F	19	English	German	Ridgefield, CT
sub012	F	27	German	English	Siegen, Germany
sub013	M	23	English	—	Monrovia, CA
sub014	F	26	English	Spanish	Riverside, CA
sub015	M	26	English	—	Edison, NJ
sub016	F	20	English	French	Gurnee, IL
sub017	F	31	English	—	Queens, NY
sub018	F	21	English	Italian	West Newbury, MA
sub019	M	26	English	—	Virginia Beach, VA
sub020	M	*Unknown*	English	Spanish	Los Angeles, CA
sub021	F	29	Korean	English	Karachi, Pakistan
sub022	M	49	Tamil	English	New Delhi, India
sub023	M	18	English	Spanish	San Clemente, CA
sub024	F	22	Vietnamese	English	Austin, TX
sub025	M	21	English	Mandarin	San Jose, CA
sub026	F	21	English	Bahasa Indonesia	Jakarta, Indonesia
sub027	F	21	English	Native Hawaiian	Kaneohe, HI
sub028	M	25	English	Hindi	Mumbai, India
sub029	M	42	Greek	English	Serres, Greece
sub030	M	28	Gujarati	English	Rajkot, India
sub031	F	20	English	—	Thousand Oaks, CA
sub032	F	21	Korean	English	Seoul, S. Korea
sub033	M	18	English	French	San Diego, CA
sub034	M	28	Telugu/Kannada	English	Bangalore, India
sub035	M	21	English	Spanish	Castro Valley, CA
sub036	F	25	English	German	Norfolk, NE
sub037	M	32	Korean	English	Seoul, S. Korea
sub038	M	30	Russian	English	Novosibirsk, Russia
sub039	M	27	Chinese	English	Shanghai, China
sub040	M	26	Korean	English	Seoul, S. Korea
sub041	F	22	English	French	Nanjing, China
sub042	F	22	English	Cantonese	Monterey Park, CA
sub043	F	28	English	Russian	Houston, TX
sub044	F	24	English	Spanish	Atlanta, GA
sub045	M	23	English	—	Los Angeles, CA
sub046	M	49	English	Spanish	Los Angeles, CA
sub047	F	18	English	—	Sacramento, CA
sub048	F	27	English	Spanish	Manhattan Beach, CA
sub049	F	29	English	—	Sacramento, CA
sub050	M	30	English	Korean	Barrington, RI
sub051	M	33	Korean	English	Seoul, S. Korea
sub052	M	26	English	German	Omaha, NE
sub053	M	29	English	—	Mill Valley, CA
sub054	F	24	English	Spanish	Oswego, IL
sub055	F	21	English	French	Cincinnati, OH
sub056	M	18	Portuguese	English	Diamond Bar, CA
sub057	M	19	English	Spanish	Abington, PA
sub058	F	28	Spanish/English	Italian	San Juan, Pueto Rico
sub059	F	22	English	Spanish	Chantilly, VA
sub060	F	22	Korean	English	San Diego, CA
sub061	F	26	Tamil	Hindi	Ooty, India
sub062	M	25	English	Twi	Tema, Ghana
sub063	F	26	English	Mandarin	Charlestown, IN
sub064	F	*Unknown*	Telugu	English	Vijayawada, India
sub065	M	19	Spanish/English	—	New York, NY
sub066	F	18	Spanish/ English	—	Los Angeles, CA
sub067	M	59	English	French	Washington, DC
sub068	M	28	Gujarati	Hindi	Junagadh, India
sub069	F	25	Mandarin	English	Suzhou, China
sub070	F	29	English	—	Brawley, CA
sub071	F	25	English	Spanish	Fresno, CA
sub072	M	36	English	—	Medina, OH
sub073	M	30	Korean	English	Iksan, S. Korea
sub074	F	30	Telugu	Hindi	Hyderabad, India
sub075	F	29	Spanish	English	Baldwin Park, CA

## References

[1] Lingala SG, Sutton BP, Miquel ME, Nayak KS (2016). Recommendations for real-time speech MRI. J. Magn. Reson. Imaging.

[2] Scott AD, Wylezinska M, Birch MJ, Miquel ME (2014). Speech MRI: Morphology and function. Phys. Medica.

[3] Ramanarayanan V (2018). Analysis of speech production real-time MRI. Comput. Speech. Lang..

[4] Hagedorn C (2019). Engineering Innovation in Speech Science: Data and Technologies. Perspect. ASHA Spec. Interes. Groups.

[5] Bresch E, Kim YC, Nayak K, Byrd D, Narayanan S (2008). Seeing speech: Capturing vocal tract shaping using real-time magnetic resonance imaging. IEEE Signal Process. Mag..

[6] Nayak, K. S., Lim, Y., Campbell-Washburn, A. E. & Steeden, J. Real-Time Magnetic Resonance Imaging. *J. Magn. Reson. Imaging*10.1002/jmri.27411 (2020). 10.1002/jmri.27411 PMC8435094 33295674

[7] Marcus DS, Wang TH, Parker J, Csernansky JG (2007). Open access series of imaging studies (OASIS): Cross-sectional MRI data in young, middle aged, nondemented, and demented older adults. J. Cogn. Neurosci..

[8] Souza R (2018). An open, multi-vendor, multi-field-strength brain MR dataset and analysis of publicly available skull stripping methods agreement. Neuroimage.

[9] Knoll F (2020). fastMRI: A Publicly Available Raw k-Space and DICOM Dataset of Knee Images for Accelerated MR Image Reconstruction Using Machine Learning. Radiol. Artif. Intell..

[10] Chen, C. *et al*. OCMR (v1.0)–Open-access multi-coil k-space dataset for cardiovascular magnetic resonance imaging. Preprint at https://arxiv.org/abs/2008.03410 (2020).

[11] Flynn, A. *et al*. Welcome to mirdata.org! http://mridata.org/ (2021).

[12] Knoll F (2020). Advancing machine learning for MR image reconstruction with an open competition: Overview of the 2019 fastMRI challenge. Magn. Reson. Med..

[13] Muckley, M. J. *et al*.. Results of the 2020 fastMRI Challenge for Machine Learning MR Image Reconstruction. *IEEE Trans. Med. Imaging*10.1109/TMI.2021.3075856 (2021). 10.1109/TMI.2021.3075856 PMC8428775 33929957

[14] Ramzi Z, Ciuciu P, Starck JL (2020). Benchmarking MRI reconstruction neural networks on large public datasets. Appl. Sci..

[15] Pezzotti, N. *et al*. An adaptive intelligence algorithm for undersampled knee MRI reconstruction. *IEEE Access* **8**, 204825–204838 (2020).

[16] Narayanan S (2014). Real-time magnetic resonance imaging and electromagnetic articulography database for speech production research (TC). J. Acoust. Soc. Am..

[17] Kim, J. *et al*. USC-EMO-MRI corpus: An emotional speech production database recorded by real-time magnetic resonance imaging. In Proc. the 10th Int. Semin. Speech Prod. 226–229 (2014).

[18] Töger J (2017). Test–retest repeatability of human speech biomarkers from static and real-time dynamic magnetic resonance imaging. J. Acoust. Soc. Am..

[19] Sorensen, T. *et al*. Database of volumetric and real-time vocal tract MRI for speech science. In *Proc. Annu. Conf. Int. Speech Commun. Assoc. (INTERSPEECH)* 645–649 (2017).

[20] Douros, I. K. *et al*. A multimodal real-time MRI articulatory corpus of French for speech research. In *Proc. Annu. Conf. Int. Speech Commun. Assoc. (INTERSPEECH)* 1556–1560 (2019).

[21] Sutton BP, Conway CA, Bae Y, Seethamraju R, Kuehn DP (2010). Faster dynamic imaging of speech with field inhomogeneity corrected spiral fast low angle shot (FLASH) at 3 T. J. Magn. Reson. Imaging.

[22] Lim Y, Lingala SG, Narayanan SS, Nayak KS (2019). Dynamic off-resonance correction for spiral real-time MRI of speech. Magn. Reson. Med..

[23] Lim Y, Bliesener Y, Narayanan SS, Nayak KS (2020). Deblurring for spiral real-time MRI using convolutional neural network. Magn. Reson. Med..

[24] Toutios A, Narayanan SS (2016). Advances in real-time magnetic resonance imaging of the vocal tract for speech science and technology research. APSIPA Trans. Signal Inf. Process..

[25] Lingala SG (2017). Feasibility of through-time spiral generalized autocalibrating partial parallel acquisition for low latency accelerated real-time MRI of speech. Magn. Reson. Med..

[26] Lingala SG (2017). A fast and flexible MRI system for the study of dynamic vocal tract shaping. Magn. Reson. Med..

[27] Niebergall A (2013). Real-time MRI of speaking at a resolution of 33 ms: Undersampled radial FLASH with nonlinear inverse reconstruction. Magn. Reson. Med..

[28] Fu M (2015). High-Resolution Dynamic Speech Imaging with Joint Low-Rank and Sparsity Constraints. Magn Reson Med.

[29] Sutton BP, Noll DC, Fessler JA (2003). Fast, iterative image reconstruction for MRI in the presence of field inhomogeneities. IEEE Trans. Med. Imaging.

[30] Fessler JA (2005). Toeplitz-Based Iterative Image Reconstruction for MRI With Correction for Magnetic Field Inhomogeneity. IEEE Trans. Signal. Process..

[31] Feng X (2018). Assessment of velopharyngeal function with multi-planar high-resolution real-time spiral dynamic MRI. Magn. Reson. Med..

[32] Lim, Y., Lingala, S. G., Toutios, A., Narayanan, S. & Nayak, K. S. Improved depiction of tissue boundaries in vocal tract real-time MRI using automatic off-resonance correction. In *Proc. Annu. Conf. Int. Speech Commun. Assoc. (INTERSPEECH)* 1765–1769, (2016).

[33] Lingala, S. G. *et al*. State-of-the-art MRI protocol for comprehensive assessment of vocal tract structure and function. In *Proc. Annu. Conf. Int. Speech Commun. Assoc. (INTERSPEECH)* 475–479 (2016).

[34] Bresch E, Nielsen J, Nayak KS, Narayanan S (2006). Synchronized and noise-robust audio recordings during realtime magnetic resonance imaging scans. J. Acoust. Soc. Am..

[35] Garofolo, J. S., Lamel, L. F., Fisher, W. M., Fiscus, J. G. & Pallett, D. S. DARPA TIMIT acoustic-phonetic continous speech corpus CD-ROM. NIST speech disc 1-1.1. *NASA STI/Recon Tech. Rep. N*, 27403 (1993).

[36] Fairbanks, F. *The Rainbow Passage. In Voice and Articulation Drillbook* 2nd edn. 124–139 (New York: Harper Row., 1960).

[37] Darley, F. L., Aronson, A. E. & Brown, J. R. *Motor Speech Disorders*. (Saunders, 1975).

[38] Smith, C. L. *Handbook of the International Phonetic Association: A guide to the use of the International Phonetic Alphabet* (Cambridge University Press, 1999).

[39] Kerr AB (1997). Real-time interactive MRI on a conventional scanner. Magn. Reson. Med..

[40] Santos, J. M., Wright, G. A. & Pauly, J. M. Flexible real-time magnetic resonance imaging framework. In *Proc. Annu. Int. Conf. IEEE Eng. Med. Biol. Soc. (EMBS)* 1048–1051 (2004). 10.1109/IEMBS.2004.1403343 17271862

[41] Narayanan SS, Nayak KS, Lee S, Sethy A, Byrd D (2004). An approach to real-time magnetic resonance imaging for speech production. J. Acoust. Soc. Am..

[42] Walsh DO, Gmitro AF, Marcellin MW (2000). Adaptive reconstruction of phased array MR imagery. Magn. Reson. Med..

[43] Burdumy M (2017). One-second MRI of a three-dimensional vocal tract to measure dynamic articulator modifications. J. Magn. Reson. Imaging.

[44] Lim Y (2019). 3D dynamic MRI of the vocal tract during natural speech. Magn. Reson. Med..

[45] Bassett EC (2014). Evaluation of highly accelerated real-time cardiac cine MRI in tachycardia. NMR Biomed..

[46] Haji-Valizadeh H (2018). Validation of highly accelerated real-time cardiac cine MRI with radial k-space sampling and compressed sensing in patients at 1.5T and 3T. Magn. Reson. Med..

[47] Steeden JA (2018). Real-time assessment of right and left ventricular volumes and function in children using high spatiotemporal resolution spiral bSSFP with compressed sensing. J. Cardiovasc. Magn. Reson..

[48] Lustig M, Donoho D, Pauly JM (2007). Sparse MRI: the application of compressed sensing for rapid MR imaging. Magn. Reson. Med..

[49] Liu B, Sebert FM, Zou Y, Ying L (2008). SparseSENSE: Randomly-Sampled Parallel Imaging using Compressed Sensing. In Proc. Int. Soc. Magn. Reson. Med. (ISMRM).

[50] Kim Y, Narayanan S, Nayak KS (2009). Accelerated three-dimensional upper airway MRI using compressed sensing. Magn. Reson. Med..

[51] Uecker M (2015). Berkeley Advanced Reconstruction Toolbox. In Proc. Int. Soc. Magn. Reson. Med. (ISMRM).

[52] Lim Y (2021). figshare.

[53] Inati SJ (2017). ISMRM Raw data format: A proposed standard for MRI raw datasets. Magn. Reson. Med..

[54] Radiological Society of North America I. CTP-The RSNA Clinical Trial Processor. *Radiological Society of North America, Inc*.

[55] Zeng DY (2019). Deep residual network for off-resonance artifact correction with application to pediatric body MRA with 3D cones. Magn. Reson. Med..

[56] Jacewicz E, Fox RA, O’Neill C, Salmons J (2009). Articulation rate across dialect, age, and gender. Lang. Var. Change.

[57] Lim Y, Narayanan S, Nayak KS (2020). Attention-gated convolutional neural networks for off-resonance correction of spiral real-time MRI. Proc. Int. Soc. Magn. Reson. Med. (ISMRM).

[58] Tian Y (2021). Aliasing artifact reduction in spiral real-time MRI. Magn. Reson. Med..

[59] Kumar P, Lim Y, Nayak KS (2021). Feasibility of super resolution speech RT-MRI using deep learning. Proc. Intl. Soc. Magn. Reson. Med. (ISMRM).

[60] Proctor, M. I., Bone, D., Katsamanis, N. & Narayanan, S. Rapid Semi-automatic Segmentation of Real-time Magnetic Resonance Images for Parametric Vocal Tract Analysis. In *Proc. Annu. Conf. Int. Speech Commun. Assoc. (INTERSPEECH)* 1576–1579 (2010).

[61] Kim, J., Kumar, N., Lee, S. & Narayanan, S. Enhanced airway-tissue boundary segmentation for real-time magnetic resonance imaging data. In *Proc. 10th Int. Semin. Speech Prod. (ISSP)* 5–8 (2014).

[62] Kim J, Toutios A, Lee S, Narayanan SS (2020). Vocal tract shaping of emotional speech. Comput. Speech Lang..

[63] Bresch E, Narayanan S (2009). Region segmentation in the frequency domain applied to upper airway real-time magnetic resonance images. IEEE Trans. Med. Imaging.

[64] Toutios, A. & Narayanan, S. S. Factor analysis of vocal-tract outlines derived from real-time magnetic resonance imaging data. in *18th International Congress of Phonetic Sciences (ICPhS)* 2015, Glasgow, UK, August 10-14, 2015

[65] Sorensen T, Toutios A, Goldstein L, Narayanan S (2019). Task-dependence of articulator synergies. J. Acoust. Soc. Am..

[66] Somandepalli, K., Toutios, A. & Narayanan, S. S. Semantic edge detection for tracking vocal tract air-tissue boundaries in real-time magnetic resonance image. In *Proc. Annu. Conf. Int. Speech Commun. Assoc. (INTERSPEECH)* 631–635 (2017).

[67] Hebbar, S. A., Sharma, R., Somandepalli, K., Toutios, A. & Narayanan, S. Vocal Tract Articulatory Contour Detection in Real-Time Magnetic Resonance Images Using Spatio-Temporal Context. *2020 IEEE Int. Conf. Acoustics, Speech and Signal Processing (ICASSP)* 7354–7358 (2020).

[68] Lammert, A. C., Proctor, M. I. & Narayanan, S. S. Data-Driven Analysis of Realtime Vocal Tract MRI using Correlated Image Regions. In *Proc. Annu. Conf. Int. Speech Commun. Assoc. (INTERSPEECH)* 1572–1575 (2010).

[69] Lammert, A., Ramanarayanan, V., Proctor, M. & Narayanan, S. Vocal tract cross-distance estimation from real-time MRI using region-of-interest analysis. In *Proc. Annu. Conf. Int. Speech Commun. Assoc. (INTERSPEECH)* 959–962 (2013).

[70] Proctor, M. *et al*. Direct estimation of articulatory kinematics from real-time magnetic resonance image sequences. In *Proc. Annu. Conf. Int. Speech Commun. Assoc. (INTERSPEECH)* 281–284 (2011).

[71] Oh M, Lee Y (2018). ACT: An Automatic Centroid Tracking tool for analyzing vocal tract actions in real-time magnetic resonance imaging speech production data. J. Acoust. Soc. Am..

[72] Toutios, A., Byrd, D., Goldstein, L. & Narayanan, S. Advances in vocal tract imaging and analysis. *The Routledge Handbook of Phonetics* (Routledge, 2019).

[73] Vaz, C., Toutios, A. & Narayanan, S. Convex hull convolutive non-negative matrix factorization for uncovering temporal patterns in multivariate time-series data. In *Proc. Annu. Conf. Int. Speech Commun. Assoc. (INTERSPEECH)* 963–967 (2016).

[74] Lammert AC, Narayanan SS (2015). On short-time estimation of vocal tract length from formant frequencies. PLoS One.

[75] Lammert AC, Shadle CH, Narayanan SS, Quatieri TF (2018). Speed-accuracy tradeoffs in human speech production. PLoS One.

[76] Alexander R, Sorensen T, Toutios A, Narayanan S (2019). A modular architecture for articulatory synthesis from gestural specification. J. Acoust. Soc. Am..

[77] Proctor, M. *et al*. Chapter 6 Studying Clicks Using Real-Time MRI. *in Click Consonants. Ch. 6* (Leiden, The Netherlands: Brill., 2020)

[78] Harper S, Goldstein L, Narayanan S (2020). Variability in individual constriction contributions to third formant values in American English /ɹ/. J. Acoust. Soc. Am..

[79] Harper, S., Goldstein, L. & Narayanan, S. L2 acquisition and production of the English rhotic pharyngeal gesture. In *Proc. Annu. Conf. Int. Speech Commun. Assoc. (INTERSPEECH)* 208–212 (2016).

[80] Monteserín, M. L., Narayanan, S. & Goldstein, L. Perceptual lateralization of coda rhotic production in Puerto Rican Spanish. In *Proc. Annu. Conf. Int. Speech Commun. Assoc. (INTERSPEECH)* 2443–2447 (2016).

[81] Oh M, Byrd D, Goldstein L, Narayanan SS (2018). Enriching the understanding of glottalic consonant production: Vertical larynx movement in Hausa ejectives and implosives. J. Acoust. Soc. Am..

[82] Oh, M., Byrd, D., Goldstein, L. & Narayanan, S. Vertical larynx actions and larynx-oral timing in ejectives and implosives. In *3rd Phonetics and Phonology in Europe (PaPE), Lecce, Italy* (2019).

[83] Proctor, M. I. *et al*. Click consonant production in Khoekhoe: a real-time MRI study. In *S*. *Shah**and**M*. *Brenzinger**(**Eds*.*)*, *Khoisan Languages and Linguistics. Proc. 5th Intl. Symposium, July 13–17, 2014, Riezlern/ Kleinwalsertal* (pp. 337–366). Cologne: Rüdiger Köppe.

[84] Lander-Portnoy M, Goldstein L, Narayanan SS (2017). Using real time magnetic resonance imaging to measure changes in articulatory behavior due to partial glossectomy. J. Acoust. Soc. Am..

[85] Hagedorn C (2017). Characterizing Articulation in Apraxic Speech Using Real-Time Magnetic Resonance Imaging. J Speech Lang Hear Res..

[86] McMicken B (2017). Bilabial Substitution Patterns during Consonant Production in a Case of Congenital Aglossia. J. Commun. Disord. Deaf Stud. Hear. Aids.

[87] Toutios A, Xu M, Byrd D, Goldstein L, Narayanan S (2020). How an aglossic speaker produces an alveolar-like percept without a functional tongue tip. J. Acoust. Soc. Am..

