# Supplementary material for: A multispeaker dataset of raw and reconstructed speech production real-time MRI video and 3D volumetric images
Source: Sci Data. 2021 Jul 20;8:187. doi: 10.1038/s41597-021-00976-x (PMC8292336; doi:10.1038/s41597-021-00976-x)
Supplement: Supplementary file 1 — Supplemental Figure 1 [file 41597_2021_976_MOESM1_ESM.pdf]

Supplemental Figure 1. References to sources of photographs corresponding to speech experiment stimuli of pictures 1-5.

**Stimuli**	**Photograph Source**
picture1	https://writefix.com/?page_id=411
picture2	https://writefix.com/?page_id=438
picture3	https://writefix.com/?page_id=443
picture4	https://writefix.com/?page_id=400
picture5	https://farmvilleherald.com/2020/03/the-worlds-biggest-man-visits-farmville/
